# Is Our Self Nothing but Reward? Neuronal Overlap and Distinction between Reward and Personal Relevance and Its Relation to Human Personality

**DOI:** 10.1371/journal.pone.0008429

**Published:** 2009-12-24

**Authors:** Björn Enzi, Moritz de Greck, Ulrike Prösch, Claus Tempelmann, Georg Northoff

**Affiliations:** 1 Department of Psychiatry, Otto-von-Guericke University Magdeburg, Magdeburg, Germany; 2 Department of Psychology, Peking University, Beijing, China; 3 Department of Neurology, Otto-von-Guericke University Magdeburg, Magdeburg, Germany; 4 Clinic for Psychosomatic Medicine and Addictive Disorders, Lübstorf, Germany; 5 Institute of Mental Health Research, University of Ottawa, Ottawa, Canada; University of Wuerzburg, Germany

## Abstract

**Background:**

The attribution of personal relevance, i.e. relating internal and external stimuli to establish a sense of belonging, is a common phenomenon in daily life. Although previous research demonstrated a relationship between reward and personal relevance, their exact neuronal relationship including the impact of personality traits remains unclear.

**Methodology/Principal Findings:**

Using functional magnetic resonance imaging, we applied an experimental paradigm that allowed us to explore the neural response evoked by reward and the attribution of personal relevance separately. We observed different brain regions previously reported to be active during reward and personal relevance, including the bilateral caudate nucleus and the pregenual anterior cingulate cortex (PACC). Additional analysis revealed activations in the right and left insula specific for the attribution of personal relevance. Furthermore, our results demonstrate a negative correlation between signal changes in both the PACC and the left anterior insula during the attribution of low personal relevance and the personality dimension novelty seeking.

**Conclusion/Significance:**

While a set of subcortical and cortical regions including the PACC is commonly involved in reward and personal relevance, other regions like the bilateral anterior insula were recruited specifically during personal relevance. Based on our correlation between novelty seeking and signal changes in both regions during personal relevance, we assume that the neuronal response to personally relevant stimuli is dependent on the personality trait novelty seeking.

## Introduction

Various imaging studies tried to clarify and to uncover the neuronal basis of our self, indicating an increased interest in this mysterious topic. In this context, a variety of different aspects and concepts of the self were investigated by neuroscientists, for instance self-recognition [1], [2], self-other discrimination [3,4], self-reflection [5] and self-relatedness [6]–[13] or more specifically reward-based self-relatedness [14], [15] and the attribution of personal relevance.

In this study, we focussed on a clearly distinguishable concept of the self, the attribution of personal relevance to everyday stimuli [14], [15] (for a recent review concerning the various concepts of the self, see [6]). Personal relevance describes the valuing of external and internal stimuli with regard to their meaning for the organism. By this, the organism establishes a sense of belongingness. [16].

The above mentioned neuroimaging studies consistently showed the involvement of a set of brain regions in different aspects and concepts of the self. These studies were able to detect various subcortical and cortical regions like the medial orbitofrontal cortex, the ventromedial prefrontal cortex (VMPFC) or the pregenual anterior cingulate cortex (PACC), the dorsomedial prefrontal, the ventrolateral prefrontal cortex, the anterior insula, the amygdala and the ventral and dorsal striatum [6], [10], [11], [13], [17]–[19] (but see also [20] and [21] for a critical position). A recent study investigated the role of the anterior insula in self-reflection [5], another different aspect of the self. The anterior insula is involved in a variety of domains, like e.g. intero- and exteroceptive awareness [22], [23] emotional salience [16] and awareness over subjective feelings [24]. By this matching function between intero-/exteroception and emotion the anterior insula could serve as a key structure in generating a sense of self [5].

The neuronal networks underlying personal relevance and reward show a strong overlap. Recent studies [14], [15] showed that regions active in a reward task like the bilateral ventral striatum, the ventral tegmental area and the VMPFC are also involved in differentiating between high and low personal relevance. In accordance with the so-called “valuation system” [25], [26], it is considered that reward has a more immediate value for the organism, whereas personal relevance mirrors a long-term value for the organism. However, the exact connection between reward and personal relevance remains unclear, and we hypothesize that reward and personal relevance can be distinguished in neuronal and psychological regard.

Personality, or more specifically temperament, makes a major contribution to human behaviour. Various brain imaging studies tried to disentangle the complex relationship between personality, its neurobiological foundations and human behaviour [27]–[29]. A widely used and reliable measurement of human personality is Cloninger's Temperament and Character Inventory (TCI; [30]). The TCI encompasses four temperament dimensions (novelty seeking, harm avoidance, reward dependence and persistence) and three character dimensions (self-directedness, co-operativeness and self-transcendence) [30]. In this study we concentrated on the temperament dimension novelty seeking (NS) and its subscales, because of its known relationship with the reward system [31]–[33]. Moreover, Cloninger himself proposed a link between NS and the mesocortical dopamine system [30], which contains key regions involved in the attribution of personal relevance like e.g. the ventral striatum and the PACC.

The general aim of our study was to investigate the behavioural and neuronal relationship between the attribution of personal relevance and reward.

First, we identified brain regions involved in both, reward processing and the attribution of personal relevance. Second, we identified specific brain regions for personal relevance. Finally, we correlated our obtained imaging data with the dimensions of Cloninger's Temperament and Character Inventory. Relying on previous research [5], [6], [16], [28], [29] we concentrated our correlation analysis on the temperament dimension novelty seeking and on brain regions crucial for the assessment of personal relevance, like the PACC and the anterior insula.

It should be noted, that this study was based on an experimental paradigm used in our previous study [14], [15]. Extending our prior work, we enlarged the sample size and included personality measures of novelty seeking in order to disentangle the complex relationship between personal relevance, reward and personality. The studies mentioned above used the so-called “functional localizer” method for comparing reward and personal relevance, i.e., the response evoked by personal relevance was always restricted on brain areas active in the reward task mirroring what the authors call “reward-based self-relatedness”. In contrast to this study, we used conjunction and exclusive masking techniques to test for both effects, i.e. reward and personal relevance, separately. This different approach allows us to show the neuronal overlap and distinction between personal relevance and reward and to link them to novelty seeking as possibly mediating personality trait.

As mentioned above, we had an a priori hypothesis concerning the involvement of the anterior insula and the pregenual anterior cingulate cortex in the attribution of personal relevance and therefore concentrated our correlation analysis on these two (anatomical) defined regions, whereas our whole brain analysis for overlapping and non-overlapping regions between reward and personal relevance is rather exploratory.

## Materials and Methods

### Ethics Statement

The presented study was approved by the institutional review board of the Department of Neurology, University of Magdeburg, Germany and by the ethical committee of the Medical Faculty, University of Magdeburg, Germany. After a detailed explanation of the study, all subjects gave their written informed consent.

### Subjects

We investigated 19 right-handed healthy subjects (12 men and 7 women, mean age 30.7 years, SD 7.1, range 23 to 50 years) without any neurological or psychiatric illness.

### Behavioral Tests

We applied different psychological tests for the behavioural characterizations of our subjects, including Beck Depression Inventory (BDI; [34]) and Cloninger's Temperament and Character Inventory (TCI; [30]) (Table S1). Further statistical analysis was carried out using repeated measurements analysis of variance (ANOVA) and dependent or independent samples *t-test*.

### Experimental Design

We applied a well-established paradigm [14], [15] that included three tasks. The reward task was a slightly modified adaptation of the task introduced by Reuter and colleagues [35], which shows a reliable activation of the reward system (Figure S3).

The experiment contained three different types of tasks. During reward trials subjects had to perform a gambling task, where they could either win or lose. During personal relevance-evaluation trials subjects indicated whether a stimulus was of high or low relevance to them. The third task was a control task in which subjects had to assess the orientation of a presented stimulus. The sequence of all trial types was designed to be as similar as possible to allow for comparison.

The whole experiment consists of eight runs (four reward runs, two personal relvance runs and two control runs) presented in a pseudo-randomized order.

All trials began with the presentation of a *decision phase* (2 s duration), where subjects were asked to bet by deciding for the left or right site of the display and had to perform a button press with either their left or right hand. During this phase a picture was displayed in the center of the screen and two small triangles at the bottom of the screen indicated which task had to be performed. The decision phase was directly followed by a *feedback phase* (2 s duration), where subjects received a short symbolized feedback. The display of the decision phase contained a symbol on the site of their response and a state bar in the center. Every location on the screen where pictures or symbols could appear was surrounded by a thin frame. Before every next trial a short *inter trial interval* (ITI, duration 1 or 2 s) was presented in which only the four empty location frames were presented.

#### Reward trials

During the decision phase of reward trials subjects were instructed to press either the left or the right button in order to gamble about amounts of their reimbursement. In the feedback phase they were informed whether they had won or lost, symbolised by a plus or a minus sign. The state bar reflected the subject's new total after the previous win or loss. Subjects were made believe that their luck during the gambling trials had direct influence on their performance however, the proportion of wins and losses was predefined and almost identical for all subjects.

#### Personal relevance trials

During the decision phase of personal relevance evaluation trials, subjects had to evaluate the presented picture and determine whether it was of high or low personal relevance. In the feedback phase of these trials an equality sign was presented when the button press was delivered in time. In contrast to both of the other tasks the minus sign was only presented when no response occurred. We decided to present an equality sign instead of the plus sign to make sure that this task had no rewarding component. The state bar was presented in these trials as well for consistency reasons. Subjects were instructed that it had no meaning and the actual value fluctuated around the midline.

#### Control trials

In the decision phase of the control trials it was the subject's task to identify the alignment of the presented picture. All stimuli had the shape of a rectangle, half of the stimuli were horizontally aligned and half of them vertically. In the feedback phase, a plus or minus sign was presented for correct and incorrect trials respectively. As in the personal relevance-evaluation task, the feedback display contained the fluctuating state bar that was irrelevant in these trials.

#### Baseline trials

After every 8 trials a baseline event occurred, in which only the four empty location frames were presented.

Each task included the presentation of all three different types of stimuli (gambling, alcohol and food stimuli) taken from the International Affective Picture System and the Normative Affective Picture System. The stimuli were chosen to maximize our ability to investigate the specific relationship between reward and personal relevance. Based on previous imaging experiments we selected stimuli that show a strong reward value such as natural reinforcers i.e. food [36], [37].

### fMRI Data Acquisition and Analysis

Functional data was collected using a 3-Tesla whole body MRI system (Siemens Trio, Erlangen, Germany) equipped with an 8-channel head coil. 32 T2*-weighted echo-planar images (EPI) per volume with blood oxygenation level-dependent (BOLD) contrast were obtained (matrix 64×64, field-of-view 224×224 mm, spatial resolution: 3,5×3,5×4 mm, TE = 30 ms, TR = 2000 ms, flip angle 80°). The slices were acquired parallel to the AC-PC plane in an odd-even interleaved acquisition order. Subjects had to complete eight scanning runs with 210 volumes per run. The first four volumes of each run were discarded.

The functional data was preprocessed and statistically analysed using the SPM2 software package (Wellcome Department of Cognitive Neuroscience, University College London, UK; http://www.fil.ion.ucl.ac.uk) and MATLAB 6.5 (The Mathworks Inc, Natick, MA, USA).

Briefly, all functional images were slice time corrected with reference to the first slice acquired, corrected for motion artifacts by realignment to a mean functional image and spatial normalized to a standard T1-weighted template provided by SPM2. The normalization was generated by warping the subject's anatomical T1-weighted scan on the T1-template and applying these parameters to all functional images [38]. The images were resampled to 2×2×2 mm and smoothed with an isotropic 6 mm full-width half-maximum Gaussian kernel. The data was high pass filtered with a frequency cut-off at 128 s.

All relevant periods (i.e. the decision phase, the feedback phase and the baseline phase) and all four conditions (reward win, reward lose, high personal and low personal relevant trials) were included in the SPM model. In each reward run five conditions are modelled (decision lose, feedback lose, decision win, feedback win and baseline), each personal relevance run also contains five conditions (decision low personal relevance, feedback low personal relevance, decision high personal relevance, feedback high personal relevance and baseline), whereas the intertrial interval was not modelled separately. For our contrasts of interest we used the feedback phase to provide as much coherence as possible to our time course analysis. A statistical model for each subject was computed by convolving a canonical response function [39], [40] with the above mentioned design. Regionally specific condition effects were tested by employing linear contrasts for each subject and different conditions. Here, we focused on the contrasts “reward win >reward lose” and “high personal relevance >low personal relevance”. The resulting contrast images were entered into a second level analysis. Here, one-sample t-test across all 19 subjects was used on images obtained for each subject's volume set and different conditions. To control for the multiple testing problem we performed a false discovery rate correction [41]. The anatomical localization of significant activations was assessed with reference to the standard stereotactic atlas by superimposition of the SPM maps on an averaged brain of all subjects.

For specification of regions only active during evaluation of personal relevance, we conducted a masking analysis implemented in SPM2 for the contrast “high personal relevance > low personal relevance” exclusively masked with “reward win > reward lose”. We thresholded the images for p<0.05 [uncorrected] for the mask and p<0.05 [FDR] for the main contrast for at least 10 contiguous voxels.

Determination of common regions for “reward win” and “high personal relevance” was calculated by a conjunction analysis implemented in SPM2 for the contrasts “reward win > reward lose” and “high personal relevance > low personal relevance”. The threshold of the resulting statistical map was p<0.001 [uncorrected] for at least 10 contiguous voxels [42].

In a second step we performed a detailed analysis based on functional and anatomical regions of interest. First, we extracted the fMRI raw data using the Marseille Region of Interest Toolbox software package MarsBaR 1.86 ([43]; http://www.sourceforge.net/projects/marsbar) relying on the functional activations obtained by our whole brain SPM analysis. Using a sphere-shaped “region of interest” (ROI, radius 5 mm) we extracted and plotted the raw signal over time for each region. Second, following the recommendations by Kriegeskorte and colleagues [44], we generated independent anatomical regions of interest using the WFU PickAtlas toolbox for SPM2 [45], [46]. For definition of the anterior insula we divided a region encompassing the whole insula (according to the AAL library [45]) into two parts following the sylvian fissure as anatomical landmark. Relying on recent literature we focused our interest on the bilateral anterior insula [5] and the pregenual anterior cingulate cortex [5]. It should be noted that the time course data obtained by this procedure for generating independent regions of interest confirms our results obtained in the above mentioned SPM analysis. This is of special importance since our conjunction results did not survive a corrected threshold. By this approach we ensured the generation of valid and independent regions for further statistical analysis using paired t-tests and Pearson-correlation [47]. Mean normalized fMRI signal values from two following time points (6 and 8 s after onset of the feedback phase) were entered into further statistical analysis. In addition, time courses of the above mentioned conditions were extracted. Statistical analysis was carried out using the software package SPSS 11.0 (SPSS Inc, Chicago).

## Results

### Behavioral Data

Reaction times during reward (mean 852.6 ms, SD 154.7) were significantly faster than during the attribution of personal relevance (mean 991.9 ms, SD 105.7) (t(18) = −4.579, p<0.001) (Figure S2).

Furthermore, we analysed the relationship between personal relevance and novelty seeking using repeated measurements analysis of variance (ANOVA). We observed a significant main effect of “task” (high vs. low personal relevance) (F(1,10) = 25.961, p<0.001) and a significant interaction between “task” (high vs. low personal relevance) and “group” (high vs. low novelty seekers) (F(1,10) = 8.746, p = 0.014).

For a more detailed analysis, we divided our study sample in three groups (low, medium and high novelty seeking) according to the lower and upper 33th percentile of the novelty seeking score and compared the reaction time between low and high novelty seeking individuals. This analysis revealed a significantly faster response during the attribution of high personal relevance in high novelty seekers compared to low novelty seekers (t(10) = 2.836, p = 0.009, independent samples *t-test*, *1-sided*). Moreover, high novelty seekers responded faster in all personal relevance events than low novelty seekers (t(10) = 1.413; *p* = 0.094, independent samples *t-test*, *1-sided*, statistical trend) (Figure S2).

### Psychological Measurements

The mean value of novelty seeking (NS) as measured by Cloninger's Temperament and Character Inventory was 19.3 (SD 6.3). For the temperament dimension novelty seeking we found a positive correlation with the character dimension self-transcendence (r[Pearson] = .506, p = 0.027), whereas we were not able to detect significant correlations between novelty seeking and the other temperament and character dimensions (see Table S2. The results of Beck's Depression Inventory (Mean: 3.0, SD 3.6) indicated the absence of a depressive mood in our study sample, thus, a possible inflation of our TCI results by depressive mood is unlikely. (Table S1).

### Neuronal Overlap between Personal Relevance and Reward

**Figure 1:**
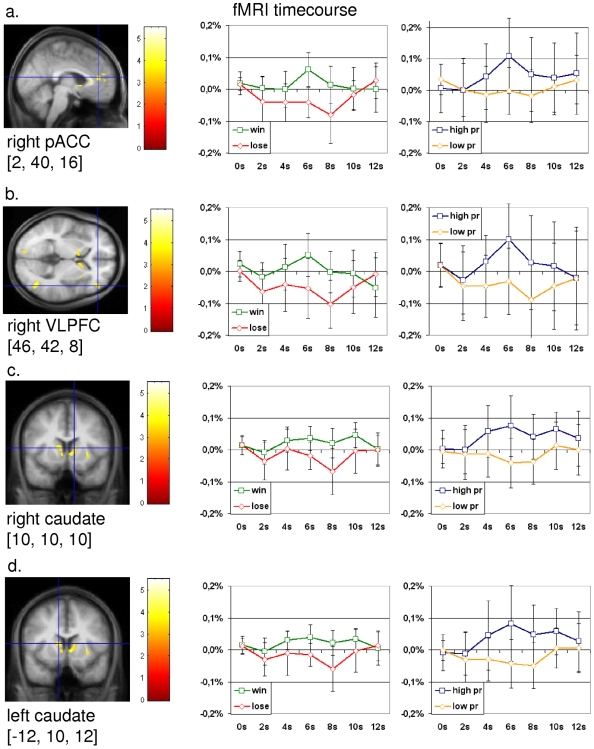
Activations and fMRI signal changes in regions derived from the SPM conjunction analysis between “high personal relevance > low personal relevance” and “win > lose”. The second level group statistic for the above mentioned contrast revealed activations in the right pregenual cingulate cortex (PACC), the right ventrolateral prefrontal cortex (VLPFC) and in the right and left caudate nucleus adjacent to the ventral striatum. The images on the far left show the statistical maps calculated with SPM2. The two diagrams in each line show the mean normalized fMRI signal changes (y-axis) for the conditions win and lose as high and low personal relevance (high pr, low pr) with t = 0 for the start of the feedback phase in healthy subjects. (*error bar: standard deviation*). a. *right PACC (2, 40, 16; z = 3.85; p[uncorr.]<0.001; k>10). b. right VLPFC (46, 42, 8; z = 3.76; p[uncorr.]<0.001; k>10). c. right caudate (10, 10, 10; z = 3.27; p[uncorr.]<0.001; k>10). d. left caudate (−12, 10, 12; z = 3.85; p[uncorr.]<0.001; k>10).*

For determination of regions active during reward and the processing of high personal relevant pictures, we performed a conjunction analysis between the contrasts “high personal relevance >low personal relevance” and “win>lose”. This approach revealed activations in the right pregenual cingulate cortex (PACC), the right and left caudate nucleus and the right ventrolateral prefrontal cortex (VLPFC) (coordinates according to the MNI stereotactical space: PACC (2, 40, 16), right caudate (10, 10, 10), left caudate (−12, 10, 12) and right VLPFC (46, 42, 8), Table 1). In these regions we observed a neuronal differentiation between “win” and “lose”, as well as between “high personal relevance” and “low personal relevance” (Figure 1). Furthermore, we observed activations in the right putamen, the right insula and the right dorsomedial prefrontal cortex.

**Table 1:** MNI coordinates of activations from the SPM contrasts.

ROI name	Contrast	coordinates [MNI]	p [FDR]	t-value	z-value
left caudate	1	−12, 10, 12	0,073	4,32	3,85
right caudate	1	10, 10, 10	0,086	3,55	3,27
right putamen	1	30, −6, −4	0,067	5,22	4,47
right VLPFC	1	46, 42, 8	0,078	4,20	3,76
right PACC (BA32)	1	2, 40, 16	0,073	4,31	3,85
right insula^3^	1	22, 24, −6	0,069	4,52	4,00
right dorsomedial PFC	1	8, 44, 48	0,071	4,46	3,95
right anterior insula	2	28, 20, 8	0,037	3,77	3,44
left anterior insula	2	−34, 26, 2	0,028	4,23	3,78
right premotor cortex	2	6, 8, 60	0,024	5,07	4,37
left Insula	2	−34, 10, 0	0,029	4,14	3,72
right supragenual ACC	2	4, 24, 24	0,035	3,87	3,51

1 conjunction of “high personal relevance > low personal relevance” with “win > lose”.

2 “high personal relevance > low personal relevance” exclusively masked with “win > lose”.

3 extending to the basal ganglia.

VLPFC: ventrolateral prefrontal cortex, PACC: pregenual anterior cingulate cortex, PFC: prefrontal cortex, ACC: anterior cingulate cortex.

### Neuronal Distinction between Personal Relevance and Reward

**Figure 2:**
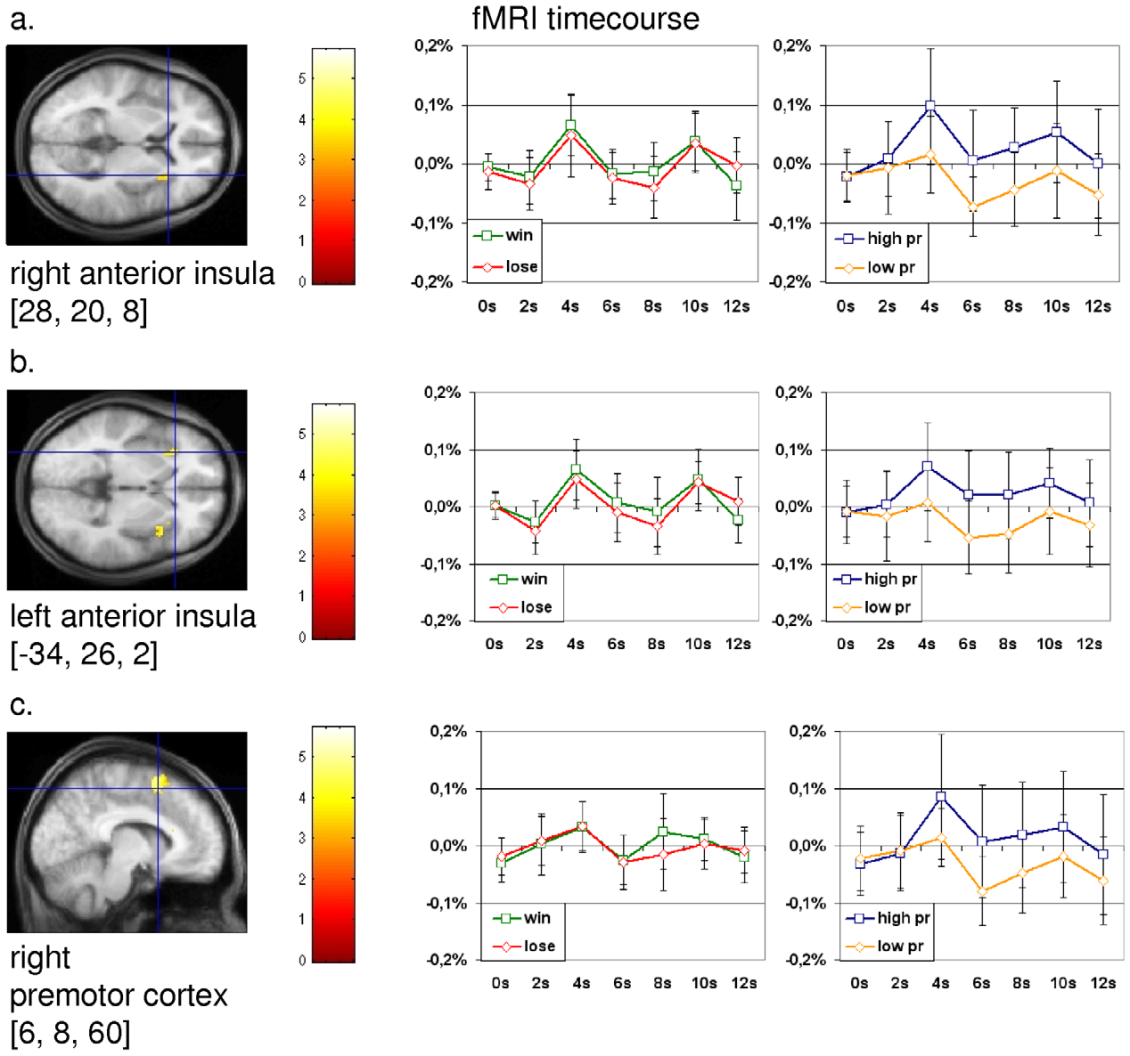
Activations and fMRI signal changes in regions derived from the contrast “(high personal relevance > low personal relevance) exclusively masked with (win > lose)”. The second level group statistic for the above mentioned contrast revealed activations in the right and left anterior insula and the right premotor cortex. The images on the far left show the statistical maps calculated with SPM2. The two diagrams in each line show the mean normalized fMRI signal changes (y-axis) for the conditions win and lose as high and low personal relevance (high pr, low pr) with t = 0 for the start of the feedback phase in healthy subjects. (error bar: standard deviation). *a. right anterior insula (28, 20, 8; z = 3.44; p[mask; uncorr]<0.05; p[FDR]<0.05; k>10). b. left anterior insula (−34, 26, 2; z = 3.78; p[mask; uncorr]<0.05; p[FDR]<0.05; k>10). c. right premotor cortex (6, 8, 60; z = 4.37; p[mask; uncorr]<0.05; p[FDR]<0.05; k>10).*

We supposed that there are also regions specific for the processing of personal relevance. For confirmation of this hypothesis, we calculated an exclusive masking analysis between the contrasts “high personal relevance > low personal relevance” and “win > lose” (Figure 2). We observed activations in the right and left anterior insula and the right premotor cortex (coordinates according to the MNI stereotactical space: right anterior insula (28, 20, 8), left anterior insula (−34, 26, 2) and right premotor cortex (6, 8, 60)).In addition, this analysis revealed an activation of the left insula (−34, 10, 0) and the right supragenual anterior cingulate cortex (4, 24, 24) (Table 1).

### Confirmation of Functional Imaging Results

**Figure 3:**
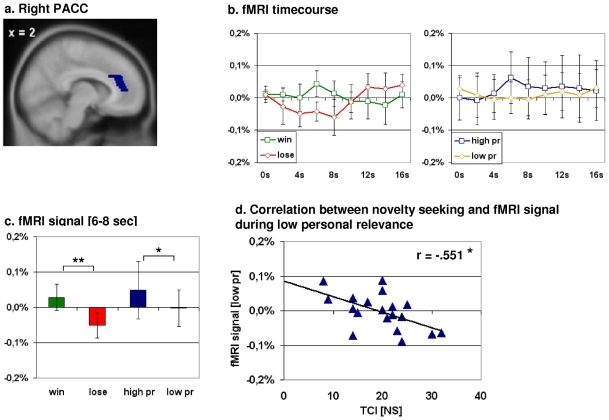
Correlation between the mean normalized fMRI signal for the time points 6 to 8 seconds derived from an anatomy-based region-of-interest and the subscale novelty-seeking (NS) of Cloninger's Temperament and Character Inventory (TCI). *a. Anatomy-based region-of-interest (ROI) encompassing the pregenual anterior cingulate cortex (PACC). b. fMRI timecourse plots derived from the pACC ROI*. The image on the left shows the neuronal differentiation between win and lose in pACC, whereas the image on the left displays the differentiation between high and low personal relevance in the very same region. *c. Mean normalized fMRI values for the time points 6 to 8 seconds*. We observed a significant differentiation between the conditions “win” and “lose” in the pACC ROI (t(18) = 6.093; *p*<0.001). As expected and in accordance with our conjunction analysis we found a significant differentiation between high and low personal relevance in the very same region (t(18) = 2.158; *p* = 0.045). *t-test for dependent variables, two-sided. Error bar: standard deviation. d. Correlation between novelty seeking and low personal relevance*. We observed a significant negative correlation between the fMRI signal change during low personal relevance and the temperament dimension novelty seeking (r[Pearson] = −.549; *p* = 0.015).

Our independent anatomically-based PACC region of interest (ROI) confirmed our above mentioned results by showing the same neuronal distinction between reward and personal relevance. In the PACC we observed a significant differentiation between “win” and “lose” (t(18) = 6.093; p<0.001), as well as between “high personal relevance” and “low personal relevance” (t(18) = 2.158; p = 0.045) (Figure 3b and 3c).

**Figure 4:**
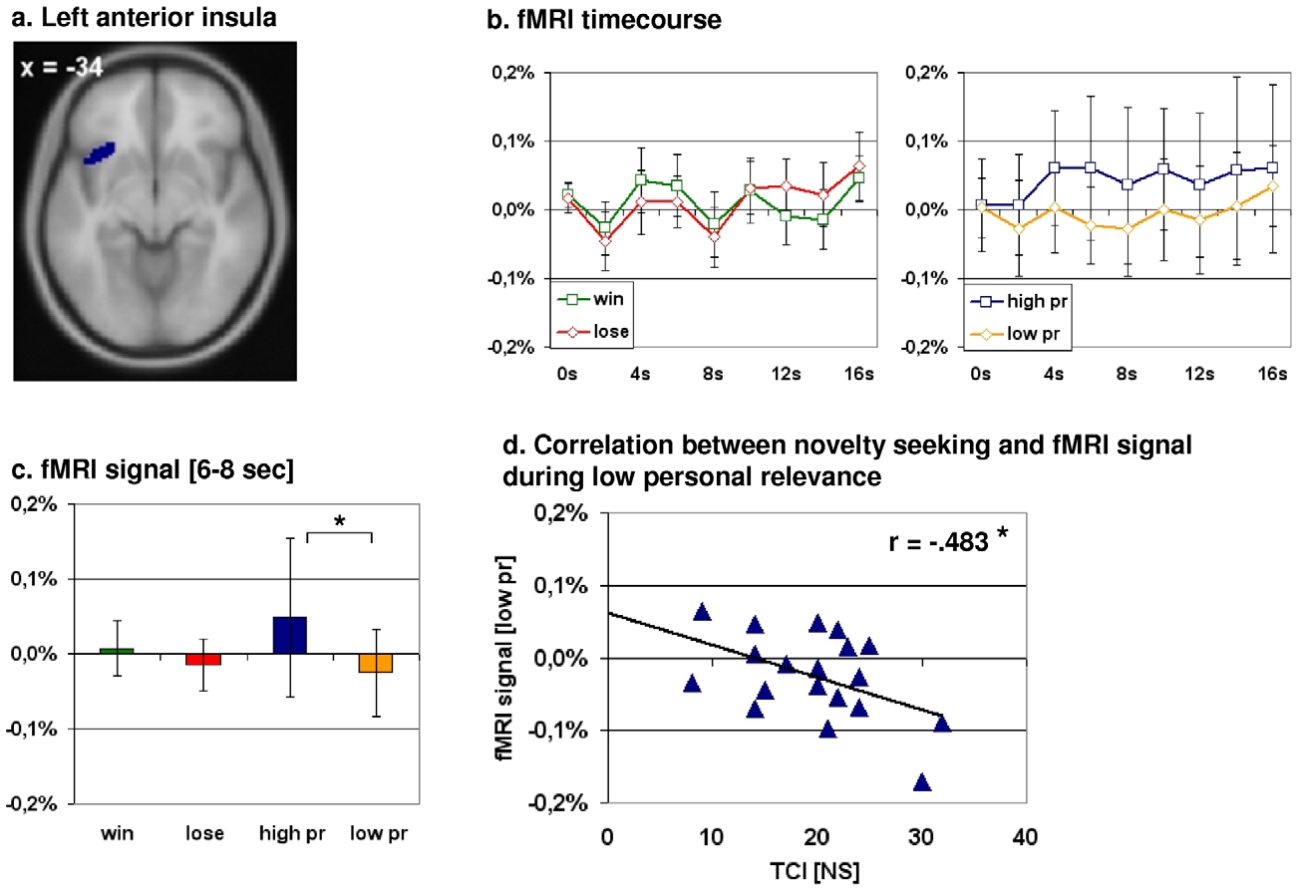
Correlation between the mean normalized fMRI signal for the time points 6 to 8 seconds derived from an anatomy-based region-of-interest and the subscale novelty seeking (NS) of Cloninger's Temperament and Character Inventory (TCI). *a. Anatomy-based region-of-interest (ROI) encompassing the left anterior insula. b. fMRI timecourse plots derived from the anterior insula ROI*. The image on the left shows the neuronal differentiation between win and lose in pACC, whereas the image on the left displays the differentiation between high and low personal relevance in the very same region. *c. Mean normalized fMRI values for the time points 6 to 8 seconds*. We observed a significant differentiation between high and low personal relevance in the left anterior insula (t(18) = 2.482; p = 0.023), whereas we were not able to detect a significant differentiation between win and (t(18) = 1.47; p = 0.159). *t test for dependent variables, two-sided. Error bar: standard deviation. d. Correlation between novelty seeking and low personal relevance*. Similar to our pACC ROI, we found a significant negative correlation between the fMRI signal change during low personal relevance and the temperament dimension novelty seeking (r[Pearson] = −.487; p = 0.035).

The independent anatomical ROI encompassing the left anterior insula also confirmed our functional imaging results. We observed a significant differentiation concerning our conditions “high personal relevance” and “low personal relevance” (t(18) = 2.482; p = 0.023). Between “win” and “lose” no significant distinction was observable (t(18) = 1.47; p = 0.159) (see Figure 4b and 4c).

Furthermore, our anatomical ROI encompassing the right anterior insula also showed a differentiation between high and low personal relevance (t(18) = 2.047; p = 0.056, statistical trend) and not between win and lose (t(18) = 0.791; p = 0.439) (Figure S4).

To confirm our findings from both the conjunction and the exclusive masking analysis, we also calculated the contrast “high personal relevance > low personal relevance” in SPM. As expected, we observed activations in the right and left ventral striatum (VS), the right and left anterior insula, the right VLPFC and the PACC. It is important to note that some of these regions are specific for personal relevance (e.g. left and right anterior insula), whereas other regions differentiate between reward and personal relevance (PACC, bilateral striatum). This observation underlines our results derived from the conjunction and masking analysis mentioned above. (For more details, see Figure S1 and Table S4).

### Personal Relevance and Novelty Seeking

To disentangle the complex relationship between personal relevance, reward and personality, we correlated the temperament dimension novelty seeking and its subscales (NS1: “exploratory excitability vs. stoic rigidity”, NS2: “impulsiveness vs. reflection”, NS3: “extravagance vs. reserve” and NS4: “disorderliness vs. regimentation”) with the functional imaging data obtained from our anatomy-based insula and PACC regions of interest.

In the case of the PACC, we found a significant negative correlation between the mean normalized fMRI signal for the condition “low personal relevance” and novelty seeking (r[Pearson] = −.551; p = 0.014) (Figure 3d and Table 2). More in detail this negative correlation was attributable to a significant negative correlation between “low personal relevance” and the subscales “impulsiveness vs. reflection” (NS2; r[Pearson] = −.538, p = 0.017) and “disorderliness vs. regimentation” (NS4; r[Pearson] = −.528, p = 0.02) (Table S3). Furthermore, no significant correlation was observed between the conditions “win”, “lose” or “high personal relevance” and other temperament dimensions different from novelty seeking (Table S5).

**Table 2:** Correlation between the dimensions of Cloninger's Temperament and Character Inventory (TCI) and the mean fMRI signal obtained for the condition “low personal relevance”.

Region	NS	HA	RD	P	SD	C	ST
Right PACC	r = −.551* *p* = 0.014	r = .19 *p* = 0.436	r = .227 *p* = 0.351	r = −.158 *p* = 0.519	r = .417(*) *p* = 0.076	r = .511* *p* = 0.025	r = −.302 *p* = 0.209
Left anterior insula	r = −.483* *p* = 0.036	r = .256 *p* = 0.289	r = .133 *p* = 0.588	r = .238 *p* = 0.326	r = −.192 *p* = 0.43	r = .13 *p* = 0.596	r = .023 *p* = 0.925

PACC: pregenual anterior cingulate cortex, NS: novelty seeking, HA: harm avoidance, RD: reward dependence, P: persistence, SD: self-directedness, C: cooperativeness, ST: self-transcendence.

Pearson correlation coefficients [r], significant correlations are labelled (**p<0.01, *p<0.05, (*)p<0.1), *two-sided*.

For the left anterior insula, correlation analysis revealed a significant negative correlation between the mean normalized fMRI signal for the condition “low personal relevance” and novelty seeking (r[Pearson] = −.483, p = 0.036) (Figure 4d). Concerning the novelty seeking subscales, only “impulsiveness vs. reflection” (NS2) showed a significant negative correlation with the attribution of low personal relevance, i.e. subjects scoring high in impulsiveness show lower fMRI signals during the evaluation of personal relevance whereas subjects scoring high in reflection exhibit a strong fMRI signal during the evaluation of low personal relevance (r[Pearson] = −.538, p = 0.036) (Table S3).

## Discussion

We here investigated the behavioural and neuronal relationship between the attribution of personal relevance, reward and personality. Our data indicate neuronal overlap between personal relevance and reward in the PACC, the bilateral caudate nucleus bordering to the ventral striatum and the right VLPFC. Neural activity in the left anterior insula was recruited specifically during personal relevance as distinguished from reward, whereas we observed a statistical trend concerning the differentiation between high and low personal relevance in the right anterior insula. Signal changes in both left anterior insula and PACC during low personal relevance correlated negatively with novelty seeking as measured by Cloninger's Temperament and Character Inventory (TCI). Taken together, these data suggest both neuronal overlap and distinction between reward and personal relevance. In addition, the neuronal response during the attribution of personal relevance is modulated by the temperament dimension of novelty seeking.

### a. Overlap between Reward, Attribution of Personal Relevance and Personality in the Anterior Cingulate Cortex

The ACC can be separated according to its main function into more a cognitive dorsal part and a rostral-ventral affective division (ACCad) [48]–[50]. Our pregenual ACC activations supposed to be involved in reward and personal relevance belongs to the affective part of the ACC (BA32, see [51]). The affective ACCad/PACC is associated with assessing salience to an object, motivational information processing, the regulation of emotional responses or emotion processing [51]–[53] and even more specifically with assigning an affective component to personally relevant stimuli [9], [16], but also in self-relatedness and personal relevance [14], [15]. In contrast, the dorsal ACC is associated with cognitive demanding tasks [50], error detection, modulation of attention, executive functions and complex motor tasks [51]. In addition, the ACCad/PACC has extensive anatomical connections to the amygdala, striatum, anterior insula and other regions [54], [55]. Taken together, these findings suggest, that the ACCad/PACC plays a key role in assigning affect to various types of tasks.

This is very well compatible especially with personal reference since any personally relevant stimulus is strongly affectively coloured be it positively or negatively [9], [16]. In the last years various brain imaging studies focussed on the anterior cingulate cortex and its relationship with personality, especially with the temperament dimension novelty seeking [27]. Magnetic resonance imaging revealed a positive correlation between harm avoidance/novelty seeking and the surface size of the ACC [56]. Measurement of the regional cerebral blood flow (rCBF) using single photon emission computed tomography (SPECT) revealed an association of novelty seeking with the activity in the ACC and the anterior insula [27].

A possible explanation for our correlation results arises from the fact, that activity in the ACC is more pronounced when external information, i.e. low relevant pictures, requires additional processing with conflicting internal states [49]. More precisely, the focussed and reflective low-NS2-scorer is confronted with a negatively coloured low personal relevant picture what leads to an increase neuronal response in the PACC in contrast to high-NS2-scorers. A recent study using magnetic resonance spectroscopy (MRS) [29] revealed a negative correlation between the glutamate level in the ACC and the sensation seeking personality trait, which is comparable to novelty seeking [29]. As explanation, the authors proposed a reduced responsiveness to negative consequences in high sensation seeking (and thus high novelty seeking) individuals caused by a reduced glutamatergic neurotransmission. According to Lisman and Grace [57], prefrontal glutamatergic excitatory activity can be interpreted as goal-related motivation and salience signal which modifies the “novelty signal” from the hippocampus. As a consequence, high novelty seekers with their reduced glutamatergic activity are not able to react adequately to salient (and probably stressful) stimuli, whereas low novelty-seekers with their pronounced analytical capacity are better able to react in an appropriate way. Moreover, this salience signal gives a possible explanation why novelty seeking specifically correlates with low personal relevance.

More indirect support comes from studies with psychiatric patients. Manic patient exhibit in elevated states an increase in novelty seeking scores [58] and therefore a tendency to impulsive reactions and difficulties in controlling anger and frustration. Concordantly, an increase in the rCBF in the ACC and the anterior insula in manic patients was observed [59].

Although there is evidence that emotion and personal relevance can be distinguished on neuronal and behavioural level [16], the exact connection between emotion, personal relevance and personality in the PACC remains unclear and should lead to further investigations.

### b. Anterior insula, Personal Relevance and Its Relation to Novelty Seeking

Our main finding concerned the specific involvement of the bilateral anterior insula in personal relevance. The insula has been involved in interoceptive awareness, emotion processing and consciousness [22], [23], [60]–[65]. Most recently, the insula has also been associated with different aspects of the self [5], [6], [16], reward [14], [15] and empathy [66]. The anterior insula receives afferents from the interoceptive system [62]–[65] and also from the extereoceptive sensory system [19]. Moreover, the insula is anatomically and functionally connected to the PACC, supporting the hypothesis that the anterior insula is crucial for linking intero-, exteroception and emotion [19], [62]–[65]. This linkage between intero- and exteroception and emotion may account for what is called here personal relevance.

This is well in accordance with a recent study [5] that showed activity in the anterior insula to be uniquely associated with self-reflection during functional MRI. Moreover, together with its well-known role in emotion processing [64], Modinos and colleagues speculate, that an emotional component is inherent to self-processing and that the sense of self is inseparable linked to emotion. This explanation is supported by the fact that psychologically an emotion is caused by the attribution of personal relevance to an event or object [67].

Our findings extend these results by showing that the insula is specifically involved in the attribution of personal relevance rather than in reward processing although both processes contain a relevant input from the brain's emotional system [68]. This is supported by the correlation pattern observed in the left anterior insula. More in detail, we noticed that individuals scoring high for novelty seeking show more deactivation during low personal relevance. Relying on resting-state fMRI studies this deactivation, i.e. the so-called “negative BOLD response” (NBR), indicates an unspecific reaction of the brain to external stimuli [69] which may be attenuated by the degree of personal relevance [16], [70]. Since novelty seeking reflects the organism's outreach towards external stimuli, one would assume a correlation with conditions that induce stronger deactivations and thus low (rather than high) personal relevance. However, this needs to be tested in the future by relating different degrees of personal relevance to external stimuli and novelty seeking.

The observed negative correlation between low personal relevance and novelty seeking was mainly based on the subscale NS2: “impulsiveness vs. reflection”. Individuals high scoring on NS2 are described as excitable, dramatic and impulsive, whereas individuals low scoring on NS2 are described as thoughtful, analytic and focussed. This is well in accordance with our behavioural results. People with high NS scores showed faster reaction times and (following the correlation) higher degrees of deactivation, so both behavioural and neuronal measurements indicate strong reagibility to external stimuli. In contrast, people with low NS scores show slower reaction times and a more pronounced activation in the left anterior insula. Since our data were mainly based on the differences in the NS2, such differential reagibility may be related to the above described psychological profiles of high and low NS2.

Although our masking analysis revealed the bilateral anterior as specific for personal relevance, it should be noted that our results concerning the right anterior insula are ambiguous. For instance, we were only able to observe a statistical trend for the differentiation between high and low personal relevance in the right anterior insula.

### c. Constitution of Value, Personal Relevance and Subcortical Regions

Relying on a conjunction analysis, we observed recruitment of the PACC, the bilateral caudate, the right VLPFC, the right putamen and the right DMPFC during reward and personal relevance. This is consistent with studies reporting involvement of these regions in both reward [26], [71]–[75] and attribution of personal relevance [14], [15] or self-relatedness ([6], [8], [9], [16], [49].

Especially subcortical regions like the bilateral caudate and the putamen seem to play an important role in the overlap between reward and personal relevance. Being part of the reward system [72], the caudate has extensive connections to different brain regions [55]. Being in accordance with a recent meta-analysis of imaging studies [54], the left caudate was co-activated, among other regions, with the right rostral anterior cingulate cortex and right medial prefrontal cortex (MPFC, BA32) extending to the left. This close functional and anatomical relationship between subcortical “reward regions” and the ACC/MPFC plays an important role in linking reward to personal relevance. Moreover, we observed activations in the caudate mainly in its dorsal part as defined by Di Martino and colleagues [55]. The dorsal caudate nucleus is supposed to be crucial for associating “reward to action” [76], [77]. In accordance with our results, we would suppose that the caudate in more general is crucial for associating stimulus relevance (immediate, i.e. reward, *and* long-term relevance) to action.

These regions are part of the so-called “valuation system” [25], [26]. This “valuation system” does not only code the stimuli's immediate relevance, i.e. the reward value, but also their long-term value for the organism. This long-term value has been associated with self-relatedness [2], [6]–[8,10,13,15,17] and the closely related concept of personal relevance [14], [15]. Moreover, the attribution of personal relevance can be considered a more stable and continuous “long-term evaluation system” when compared to reward. Further support for this hypothesis arises from the observation that the reaction times are significantly longer during the evaluation of personal relevance when compared to reward.

4. Schilbach L, Eickhoff S, Rotarska-Jagiela A, Fink G, Vogeley K (2008) Minds at rest? Social cognition as the default mode of cognizing and its putative relationship to the “default system” of the brain. Conscious Cogn 17: 457-467.

## Supporting Information

Figure S1 Contrast “(high personal reference) > (low personal reference)” Activations and fMRI signal changes in regions derived from the contrast “high personal relevance > low personal relevance”. The images on the far left show the t-contrast calculated with SPM2. The two diagrams in each line show the mean normalized fMRI signal changes (y-axis) for the conditions win and lose as high and low personal relevance (high pr, low pr) with t = 0 for the start of the feedback phase in healthy subjects. (error bar: standard deviation) The second level group statistic for the above mentioned contrast revealed activations in the right (10, 8, 2; z = 3.70; p[FDR]<0.01; k>20) and left (−8, 8, 4; z = 4.05; p[FDR]<0.01; k>20) ventral striatum (VS), the right (36, 28, 4; z = 4.57; p[FDR]<0.01; k>20) and left (−38, 16, −2; z = 5.23; p[FDR]<0.01; k>20) anterior insula, the right ventrolateral prefrontal cortex (VLPFC) (44, 36, 10; z = 4.16; p[FDR]<0.01; k>20) and the pregenual cingulate cortex (PACC) (0, 40, 16; z = 3.94; p[FDR]<0.01; k>20). As expected, we observe activations in regions specific for the differentiation between high and low personal relevance like e.g. the bilateral anterior insula. Moreover, the bilateral VS, the VLPFC and the PACC show a differentiation in both domains, reward and personal relevance. This supports our proposed model for neuronal integration and differentiation between reward and personal relevance.
(1.31 MB TIF)

Figure S2 Reaction time during personal relevance in high and low novelty seeking individuals Reaction time in high and low novelty seeking individuals After division of our study population in three groups (high NS (n = 6): mean 25.3 (SD 3.7), medium NS (n = 7): mean 20.0 (SD 1.7), low NS (n = 6): mean 12.3 (SD 3.0)), we compared the average reaction time for reward (win and lose) and personal relevance (high and low personal relevance). Concerning the reward task, we observed no significant difference between high and low novelty seekers (t(10) = 0.611; p = 0.277), whereas in the personal relevance task high novelty seekers responded faster than low novelty seekers (t(10) = 1.413; p = 0.094, statistical trend). t-test for independent variables, 1-sided Error bar: standard deviation
(0.03 MB TIF)

Figure S3 Schematic illustration of the paradigm used in this study.
(0.39 MB TIF)

Figure S4 fMRI results for the right anterior insula a. fMRI signal changes in the right anterior insula. The two diagrams show the mean normalized fMRI signal changes (y-axis) for the conditions win and lose and high and low personal relevance (high pr, low pr) with t = 0 for the start of the feedback phase in healthy subjects. (error bar: standard deviation) b. mean normalized fMRI signal for the timepoints 6 to 8 sec after the beginning of the feedback phase. The mean normalized fMRI signal indicates a statistical trend for the differentiation between the conditions “high personal relevance” and “low personal relevance” (t(18) = 2.047; p = 0.056) in the right anterior insula, whereas we were not able to observe a significant differentiation between “win” and “lose” (t(18) = 0.791; p = 0.439). t-test for paired variables, 2-sided Error bar: standard deviation
(0.46 MB TIF)

Table S1 Characteristics of the study population. mean (Standard deviation) Abbreviations: MWT-A (german: Mehrfachwortschatzintelligenztest [1]):Measurement of the general intelligence level, LPS-3 (german: Leistungsprüfsystem [2]): Measurement of the general intelligence level, BDI: Beck Depression Inventory [3], german version, TCI: Cloninger's Temperament and Character Inventory [4] and its dimensions and subscales (NS: novelty seeking, HA: harm avoidance, RD: reward dependence, P: persistence, SD: self-directedness, C: cooperativeness, ST: self-transcendence, NS1: novelty seeking subscale 1 - exploratory excitability vs. stoic rigidity, NS2: novelty seeking subscale 2 - impulsiveness vs. reflection, NS3: novelty seeking subscale 3 - extravagance vs. reserve, NS4: novelty seeking subscale 4 - disorderliness vs. regimentation) References: 1. Lehrl S, Merz J, Burkhard G, Fischer B (1991) Mehrfach-Wortschatz-Intelligenztest (MWT). Erlangen: Perimed-Fachbuch-Verlag. 2. Horn W (1983) L-P-S Leistungsprüfsystem. Göttingen: Hogrefe Verlag. 3. Hautzinger M, Bailer M, Worall H, Keller F (1995) Beck-Depressions-Inventar. Bern: Hans Huber. 4. Cloninger C, Przybeck T, Svrakic D, Wetzel R (1999) Das Temperament- und Charakter-Inventar TCI. Frankfurt: Sweets & Zeitlinger.
(0.04 MB DOC)

Table S2 Correlation between the dimensions of Cloninger's Temperament and Character Inventory (n = 19). Pearson correlation coefficients [r], significant correlations are labelled (**p<0.01, *p<0.05, (*)p<0.1), two-sided Abbreviations: NS: novelty seeking, HA: harm avoidance, RD: reward dependence, P: persistence, SD: self-directedness, C: cooperativeness, ST: self-transcendence
(0.03 MB DOC)

Table S3 Correlation between the subscales of the temperament dimensions novelty seeking and the mean fMRI signal (6 to 8 seconds) obtained for the condition low personal relevance. Pearson correlation coefficients [r], significant correlations are labelled (**p<0.01, *p<0.05, (*)p<0.1), two-sided Abbreviations: NS1: novelty seeking subscale 1 - exploratory excitability vs. stoic rigidity, NS2: novelty seeking subscale 2 - impulsiveness vs. reflection, NS3: novelty seeking subscale 3 - extravagance vs. reserve, NS4: novelty seeking subscale 4 - disorderliness vs. regimentation PACC: pregenual anterior cingulate cortex
(0.03 MB DOC)

Table S4 MNI coordinates of activations for the contrast. Abbreviations: VLPFC: ventrolateral prefrontal cortex, IFG: inferior frontal gyrus, ACC: anterior cingulated cortex, DMPFC: dorsomedial prefrontal cortex, SMA: supplementary motor area, BA32: Brodman Area 32
(0.04 MB DOC)

Table S5 Correlation between the different temperament dimensions and the mean fMRI signal (6 to 8 seconds) obtained for the conditions high personal relevance, low personal relevance, win and lose. Pearson correlation coefficients [r], significant correlations are labelled (**p<0.01, *p<0.05, (*)p<0.1), two-sided Abbreviations: NS: novelty seeking, HA: harm avoidance, RD: reward dependence, P: persistence, PACC: pregenual anterior cingulate cortex
(0.04 MB DOC)
