# Supplementary figures and images for: Is Our Self Nothing but Reward? Neuronal Overlap and Distinction between Reward and Personal Relevance and Its Relation to Human Personality
Source: PLoS One. 2009 Dec 24;4(12):e8429. doi: 10.1371/journal.pone.0008429 (PMC2794541; doi:10.1371/journal.pone.0008429)

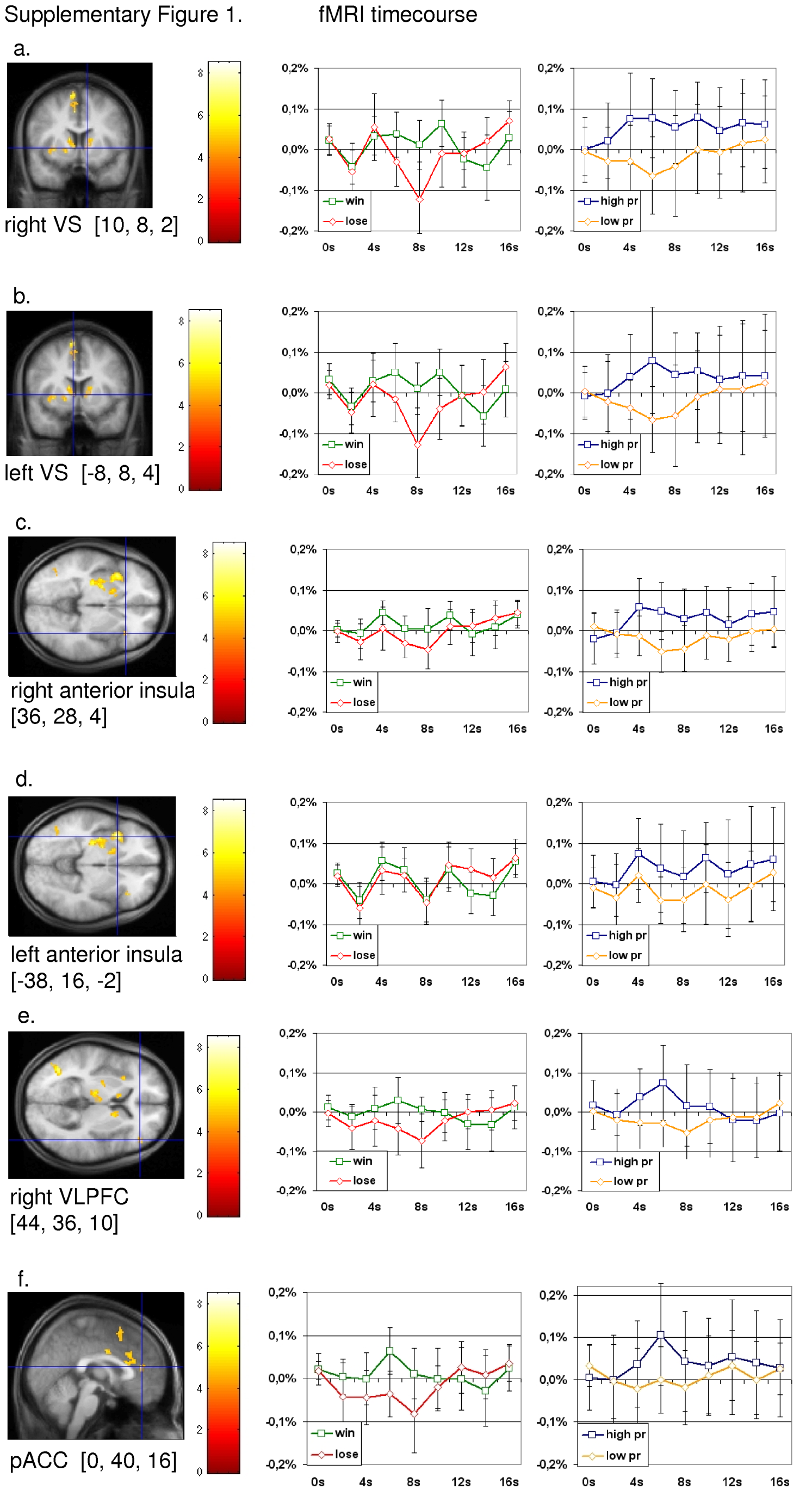

Supplement: Figure S1 — Contrast “(high personal reference) > (low personal reference)” Activations and fMRI signal changes in regions derived from the contrast “high personal relevance > low personal relevance”. The images on the far left show the t-contrast calculated with SPM2. The two diagrams in each line show the mean normalized fMRI signal changes (y-axis) for the conditions win and lose as high and low personal relevance (high pr, low pr) with t = 0 for the start of the feedback phase in healthy subjects. (error bar: standard deviation) The second level group statistic for the above mentioned contrast revealed activations in the right (10, 8, 2; z = 3.70; p[FDR]<0.01; k>20) and left (−8, 8, 4; z = 4.05; p[FDR]<0.01; k>20) ventral striatum (VS), the right (36, 28, 4; z = 4.57; p[FDR]<0.01; k>20) and left (−38, 16, −2; z = 5.23; p[FDR]<0.01; k>20) anterior insula, the right ventrolateral prefrontal cortex (VLPFC) (44, 36, 10; z = 4.16; p[FDR]<0.01; k>20) and the pregenual cingulate cortex (PACC) (0, 40, 16; z = 3.94; p[FDR]<0.01; k>20). As expected, we observe activations in regions specific for the differentiation between high and low personal relevance like e.g. the bilateral anterior insula. Moreover, the bilateral VS, the VLPFC and the PACC show a differentiation in both domains, reward and personal relevance. This supports our proposed model for neuronal integration and differentiation between reward and personal relevance. (1.31 MB TIF) [file pone.0008429.s001.tif]

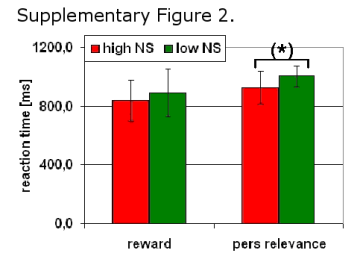

Supplement: Figure S2 — Reaction time during personal relevance in high and low novelty seeking individuals Reaction time in high and low novelty seeking individuals After division of our study population in three groups (high NS (n = 6): mean 25.3 (SD 3.7), medium NS (n = 7): mean 20.0 (SD 1.7), low NS (n = 6): mean 12.3 (SD 3.0)), we compared the average reaction time for reward (win and lose) and personal relevance (high and low personal relevance). Concerning the reward task, we observed no significant difference between high and low novelty seekers (t(10) = 0.611; p = 0.277), whereas in the personal relevance task high novelty seekers responded faster than low novelty seekers (t(10) = 1.413; p = 0.094, statistical trend). t-test for independent variables, 1-sided Error bar: standard deviation (0.03 MB TIF) [file pone.0008429.s002.tif]

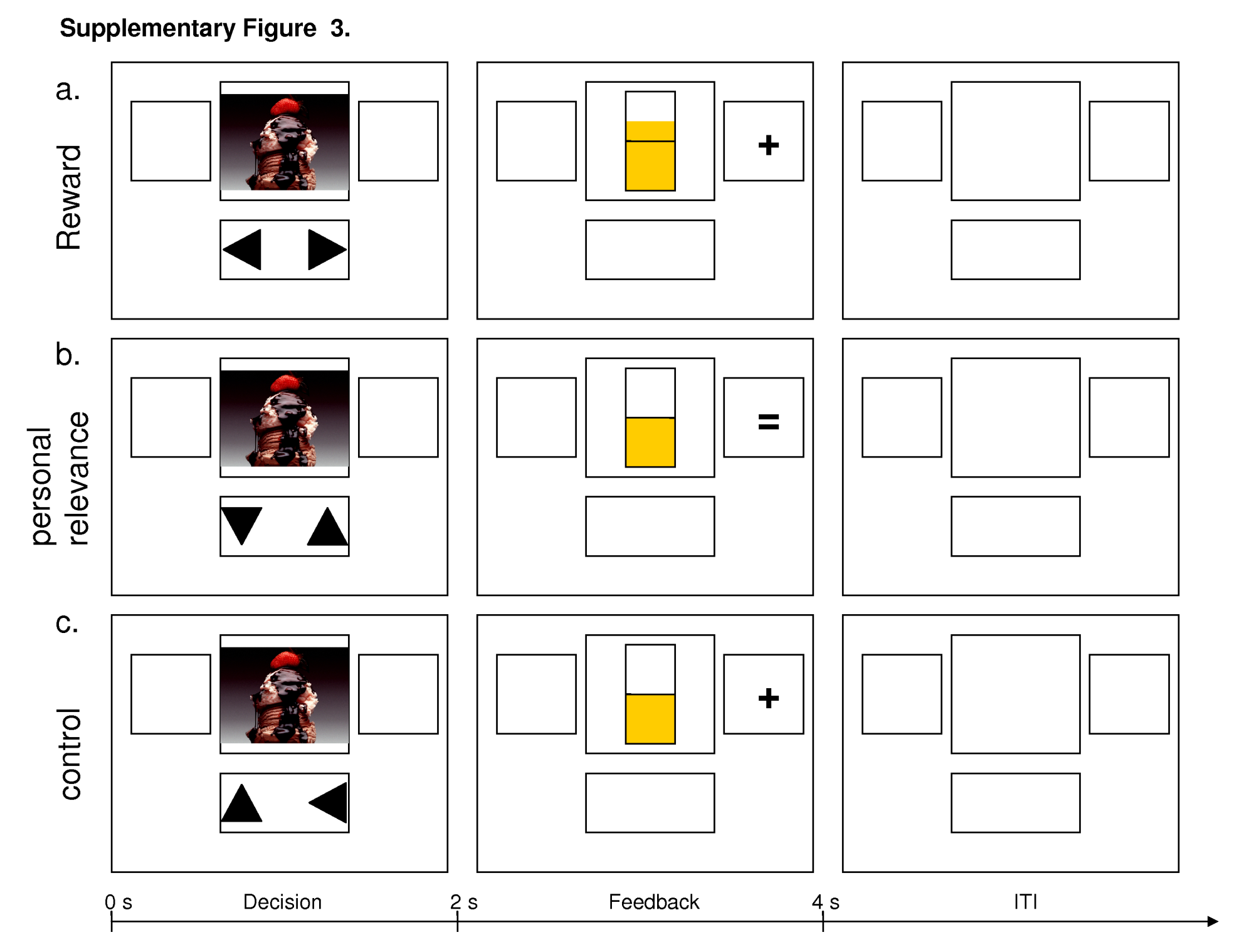

Supplement: Figure S3 — Schematic illustration of the paradigm used in this study. (0.39 MB TIF) [file pone.0008429.s003.tif]

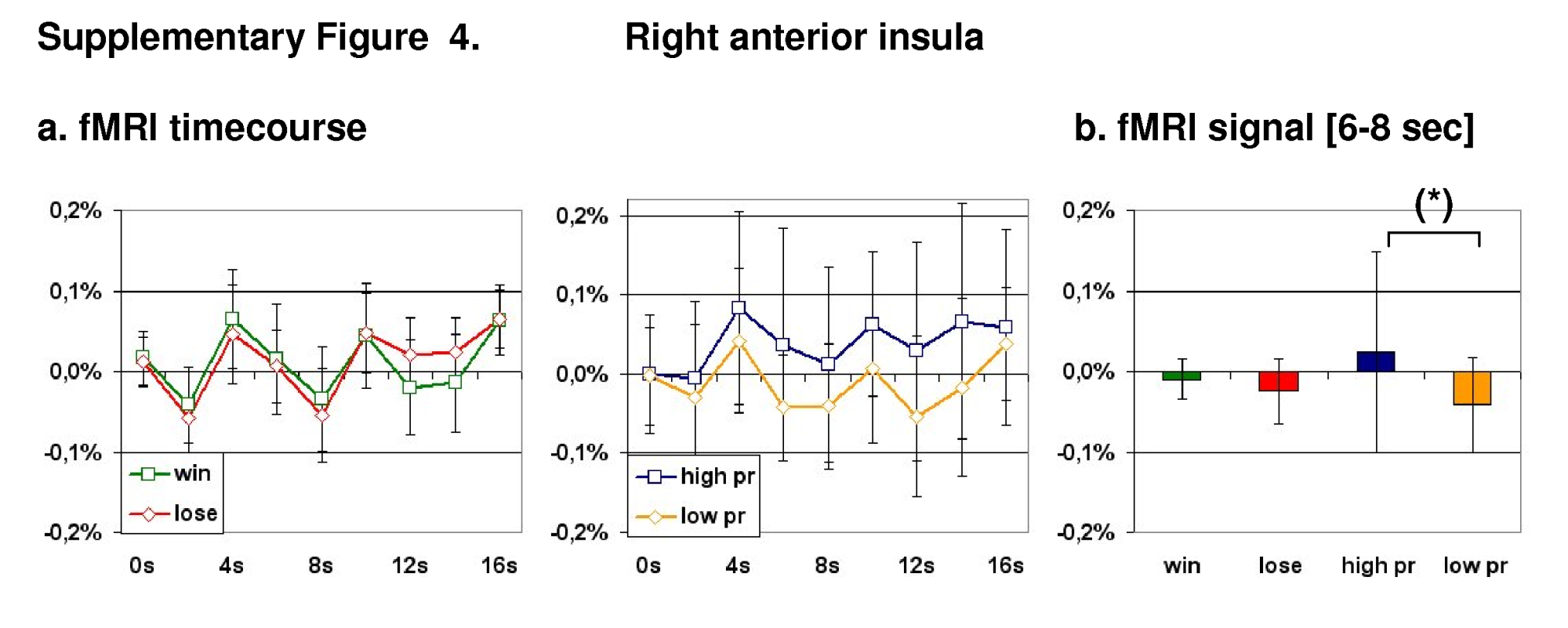

Supplement: Figure S4 — fMRI results for the right anterior insula a. fMRI signal changes in the right anterior insula. The two diagrams show the mean normalized fMRI signal changes (y-axis) for the conditions win and lose and high and low personal relevance (high pr, low pr) with t = 0 for the start of the feedback phase in healthy subjects. (error bar: standard deviation) b. mean normalized fMRI signal for the timepoints 6 to 8 sec after the beginning of the feedback phase. The mean normalized fMRI signal indicates a statistical trend for the differentiation between the conditions “high personal relevance” and “low personal relevance” (t(18) = 2.047; p = 0.056) in the right anterior insula, whereas we were not able to observe a significant differentiation between “win” and “lose” (t(18) = 0.791; p = 0.439). t-test for paired variables, 2-sided Error bar: standard deviation (0.46 MB TIF) [file pone.0008429.s004.tif]
