# Supplementary material for: Is Our Self Nothing but Reward? Neuronal Overlap and Distinction between Reward and Personal Relevance and Its Relation to Human Personality
Source: PLoS One. 2009 Dec 24;4(12):e8429. doi: 10.1371/journal.pone.0008429 (PMC2794541; doi:10.1371/journal.pone.0008429)
Supplement: Table S1 — Characteristics of the study population. mean (Standard deviation) Abbreviations: MWT-A (german: Mehrfachwortschatzintelligenztest [1]):Measurement of the general intelligence level, LPS-3 (german: Leistungsprüfsystem [2]): Measurement of the general intelligence level, BDI: Beck Depression Inventory [3], german version, TCI: Cloninger's Temperament and Character Inventory [4] and its dimensions and subscales (NS: novelty seeking, HA: harm avoidance, RD: reward dependence, P: persistence, SD: self-directedness, C: cooperativeness, ST: self-transcendence, NS1: novelty seeking subscale 1 - exploratory excitability vs. stoic rigidity, NS2: novelty seeking subscale 2 - impulsiveness vs. reflection, NS3: novelty seeking subscale 3 - extravagance vs. reserve, NS4: novelty seeking subscale 4 - disorderliness vs. regimentation) References: 1. Lehrl S, Merz J, Burkhard G, Fischer B (1991) Mehrfach-Wortschatz-Intelligenztest (MWT). Erlangen: Perimed-Fachbuch-Verlag. 2. Horn W (1983) L-P-S Leistungsprüfsystem. Göttingen: Hogrefe Verlag. 3. Hautzinger M, Bailer M, Worall H, Keller F (1995) Beck-Depressions-Inventar. Bern: Hans Huber. 4. Cloninger C, Przybeck T, Svrakic D, Wetzel R (1999) Das Temperament- und Charakter-Inventar TCI. Frankfurt: Sweets & Zeitlinger. (0.04 MB DOC) [file pone.0008429.s005.doc]

**Supplementary Table S1: Characteristics of the study population**

|  | healthy |
| --- | --- |
| *n* | 19 |
| age (years) | 30.7 (7.1) |
| gender (m/f) | 12/7 |
| MWT-A | 121.4 (12.3) |
| LPS-3 | 111.9 (17.8) |
| BDI | 3.3 (3.6) |
| TCI: NS | 19.3 (6.3) |
| TCI: HA | 12.6 (3.8) |
| TCI: RD | 16.1 (3.5) |
| TCI: P | 4.3 (1.7) |
| TCI: SD | 34.1 (5.2) |
| TCI: C | 32.2 (6.2) |
| TCI: ST | 9.5 (6.9) |
| TCI: NS1 | 7.1 (2.4) |
| TCI: NS2 | 4.2 (2.2) |
| TCI: NS3 | 5.1 (2.3) |
| TCI: NS4 | 3.7 (1.9) |

mean (Standard deviation)

**Abbreviations**: MWT-A (*german: Mehrfachwortschatzintelligenztest [1])*:Measurement of the general intelligence level, LPS-3 *(german: Leistungsprüfsystem [2])*: Measurement of the general intelligence level, BDI: Beck Depression Inventory [3], german version, TCI: Cloninger’s Temperament and Character Inventory [4] and its dimensions and subscales (NS: novelty seeking, HA: harm avoidance, RD: reward dependence, P: persistence, SD: self-directedness, C: cooperativeness, ST: self-transcendence, NS1: novelty seeking subscale 1 – exploratory excitability vs. stoic rigidity, NS2: novelty seeking subscale 2 – impulsiveness vs. reflection, NS3: novelty seeking subscale 3 – extravagance vs. reserve, NS4: novelty seeking subscale 4 – disorderliness vs. regimentation)

**References**:

1. Lehrl S, Merz J, Burkhard G, Fischer B (1991) Mehrfach-Wortschatz-Intelligenztest (MWT). Erlangen: Perimed-Fachbuch-Verlag.

2. Horn W (1983) L-P-S Leistungsprüfsystem. Göttingen: Hogrefe Verlag.

3. Hautzinger M, Bailer M, Worall H, Keller F (1995) Beck-Depressions-Inventar. Bern: Hans Huber.

4. Cloninger C, Przybeck T, Svrakic D, Wetzel R (1999) Das Temperament- und Charakter-Inventar TCI. Frankfurt: Sweets & Zeitlinger.
