# Supplementary material for: Is Our Self Nothing but Reward? Neuronal Overlap and Distinction between Reward and Personal Relevance and Its Relation to Human Personality
Source: PLoS One. 2009 Dec 24;4(12):e8429. doi: 10.1371/journal.pone.0008429 (PMC2794541; doi:10.1371/journal.pone.0008429)
Supplement: Table S2 — Correlation between the dimensions of Cloninger's Temperament and Character Inventory (n = 19). Pearson correlation coefficients [r], significant correlations are labelled (**p<0.01, *p<0.05, (*)p<0.1), two-sided Abbreviations: NS: novelty seeking, HA: harm avoidance, RD: reward dependence, P: persistence, SD: self-directedness, C: cooperativeness, ST: self-transcendence (0.03 MB DOC) [file pone.0008429.s006.doc]

| NS | 1 |  |  |  |  |  |  |
| --- | --- | --- | --- | --- | --- | --- | --- |
| HA | r = -.3  *p*= 0.211 | 1 |  |  |  |  |  |
| RD | r = .144  *p*= 0.555 | r = -.055  *p* = 0.822 | 1 |  |  |  |  |
| P | r = .056  *p* = 0.82 | r = -.056  *p* = 0.82 | r = -.193  *p*= 0.429 | 1 |  |  |  |
| SD | r = -.198  *p*= 0.417 | r = .042  *p*= 0.864 | r = .18  *p*= 0.461 | r= .482*  *p*=0.037 | 1 |  |  |
| C | r = -.26  *p*= 0.283 | r = .24  *p*= 0.322 | r = .68**  *p*= 0.001 | r=.552*  *p*=0.014 | r = .471*  *p*= 0.042 | 1 |  |
| ST | r = .506*  *p*= 0.027 | r = -.27  *p*= 0.263 | r = .08  *p*= 0.743 | r =.557*  *p*=0.013 | r = -.68**  *p*= 0.001 | r = -.286  *p*=0.236 | 1 |

**Supplementary Table S2: Correlation between the dimensions of Cloninger’s Temperament and Character Inventory (*n = 19*)**

Pearson correlation coefficients [r], significant correlations are labelled

(***p* < 0.01, **p* < 0.05, (*)p<0.1), *two-sided*

**Abbreviations**: NS: novelty seeking, HA: harm avoidance, RD: reward dependence, P: persistence, SD: self-directedness, C: cooperativeness, ST: self-transcendence
