# Supplementary material for: Is Our Self Nothing but Reward? Neuronal Overlap and Distinction between Reward and Personal Relevance and Its Relation to Human Personality
Source: PLoS One. 2009 Dec 24;4(12):e8429. doi: 10.1371/journal.pone.0008429 (PMC2794541; doi:10.1371/journal.pone.0008429)
Supplement: Table S3 — Correlation between the subscales of the temperament dimensions novelty seeking and the mean fMRI signal (6 to 8 seconds) obtained for the condition low personal relevance. Pearson correlation coefficients [r], significant correlations are labelled (**p<0.01, *p<0.05, (*)p<0.1), two-sided Abbreviations: NS1: novelty seeking subscale 1 - exploratory excitability vs. stoic rigidity, NS2: novelty seeking subscale 2 - impulsiveness vs. reflection, NS3: novelty seeking subscale 3 - extravagance vs. reserve, NS4: novelty seeking subscale 4 - disorderliness vs. regimentation PACC: pregenual anterior cingulate cortex (0.03 MB DOC) [file pone.0008429.s007.doc]

**Supplementary Table S3: Correlation between the subscales of the temperament dimensions novelty seeking and the mean fMRI signal (6 to 8 seconds) obtained for the condition low personal relevance**

|  | Region | NS | NS1 | NS2 | NS3 | NS4 |
| --- | --- | --- | --- | --- | --- | --- |
| low personal relevance | Right PACC | r = -.551*  *p* = 0.014 | r = .031  *p* = 0.9 | r = -.538*  *p* = 0.017 | r = -.362  *p* = 0.128 | r = -.528*  *p* = 0.02 |
| low personal relevance | Left anterior insula | r = -.483*  *p* = 0.036 | r = -.149  *p* = 0.541 | r= -.702**  *p* = 0.001 | r = -.353  *p* = 0.138 | r = -.202  *p* = 0.408 |

Pearson correlation coefficients [r], significant correlations are labelled

(***p* < 0.01, **p* < 0.05, (*)p<0.1), *two-sided*

**Abbreviations**: NS1: novelty seeking subscale 1 – exploratory excitability vs. stoic rigidity, NS2: novelty seeking subscale 2 – impulsiveness vs. reflection, NS3: novelty seeking subscale 3 – extravagance vs. reserve, NS4: novelty seeking subscale 4 – disorderliness vs. regimentation

PACC: pregenual anterior cingulate cortex
