# Supplementary material for: Is Our Self Nothing but Reward? Neuronal Overlap and Distinction between Reward and Personal Relevance and Its Relation to Human Personality
Source: PLoS One. 2009 Dec 24;4(12):e8429. doi: 10.1371/journal.pone.0008429 (PMC2794541; doi:10.1371/journal.pone.0008429)
Supplement: Table S4 — MNI coordinates of activations for the contrast. Abbreviations: VLPFC: ventrolateral prefrontal cortex, IFG: inferior frontal gyrus, ACC: anterior cingulated cortex, DMPFC: dorsomedial prefrontal cortex, SMA: supplementary motor area, BA32: Brodman Area 32 (0.04 MB DOC) [file pone.0008429.s008.doc]

**Supplementary Table S4: MNI coordinates of activations for the contrast "(high personal reference) > (low personal reference)"**

**Abbreviations**: VLPFC: ventrolateral prefrontal cortex, IFG: inferior frontal gyrus, ACC: anterior cingulated cortex, DMPFC: dorsomedial prefrontal cortex, SMA: supplementary motor area, BA32: Brodman Area 32

| ROI name | coordinates  [MNI] | p [FDR] | t-value | z-value |
| --- | --- | --- | --- | --- |
| right ventral striatum | 10, 8, 2 | 0.009 | 4.62 | 3.70 |
| left ventral striatum | -8, 8, 4 | 0.007 | 5.28 | 4.05 |
| right anterior insula | 36, 28, 4 | 0.003 | 6.41 | 4.57 |
| left anterior insula | -38, 16, -2 | 0.002 | 8,22 | 5.23 |
| right VLPFC/ IFG | 44, 36, 10 | 0.006 | 5.50 | 4.16 |
| pregenual ACC | 0, 40, 16 | 0.007 | 5.07 | 3.94 |
| left thalamus | -12, -22, 10 | 0.003 | 6.91 | 4.77 |
| left putamen | -20, 10, 0 | 0.006 | 5.41 | 4.12 |
| left supragenual ACC | -4, 28, 24 | 0.005 | 5.70 | 4.25 |
| left medial temporal gyrus | -44, -66, 8 | 0.002 | 7.44 | 4.97 |
| left inferior parietal gyrus | -58, -32, 50 | 0.003 | 6.52 | 4.61 |
| left superior frontal gyrus | -14, 40, 42 | 0.002 | 8.50 | 5.32 |
| left caudate | -12, 12, 4 | 0.005 | 5.59 | 4.20 |
| DMPFC | -8, 42, 44 | 0.004 | 5.95 | 4.37 |
| left SMA/ BA32 | -6, 8, 48 | 0.005 | 5.64 | 4.23 |
| right cuneus | 18, -92, 12 | 0.008 | 5.02 | 3.92 |
| left fusiform gyrus | -38, -72, -14 | 0.004 | 6.21 | 4.48 |
| right cerebellum | 10, -52, -10 | 0.002 | 7.80 | 5.09 |
