# Supplementary material for: Is Our Self Nothing but Reward? Neuronal Overlap and Distinction between Reward and Personal Relevance and Its Relation to Human Personality
Source: PLoS One. 2009 Dec 24;4(12):e8429. doi: 10.1371/journal.pone.0008429 (PMC2794541; doi:10.1371/journal.pone.0008429)
Supplement: Table S5 — Correlation between the different temperament dimensions and the mean fMRI signal (6 to 8 seconds) obtained for the conditions high personal relevance, low personal relevance, win and lose. Pearson correlation coefficients [r], significant correlations are labelled (**p<0.01, *p<0.05, (*)p<0.1), two-sided Abbreviations: NS: novelty seeking, HA: harm avoidance, RD: reward dependence, P: persistence, PACC: pregenual anterior cingulate cortex (0.04 MB DOC) [file pone.0008429.s009.doc]

**Supplementary Table S5: Correlation between the different temperament dimensions and the mean fMRI signal (6 to 8 seconds) obtained for the conditions high personal relevance, low personal relevance, win and lose.**

| Condition | Region | NS | HA | RD | P |
| --- | --- | --- | --- | --- | --- |
| high personal relevance | Right PACC | r = -.059  *p* = 0.81 | r = -.194  *p* = 0.426 | r = .058  *p* = 0.813 | r = -.331  *p* = 0.166 |
| high personal relevance | Left anterior insula | r = .078  *p* = 0.751 | r = -.138  *p* = 0.572 | r = .042  *p* = 0.866 | r = -.33  *p* = 0.168 |
| low personal relevance | Right PACC | r = -.551*  *p* = 0.014 | r = .171  *p* = 0.483 | r = .22  *p* = 0.364 | r = -.164  *p* = 0.014 |
| low personal relevance | Left anterior insula | r = -.483*  *p* = 0.036 | r = .246  *p* = 0.31 | r = .128  *p* = 0.602 | r = .236  *p* = 0.330 |
| win | Right PACC | r = .02  *p* = 0.934 | r = .058  *p* = 0.814 | r = .061  *p* = 0.804 | r = .385  *p* = 0.104 |
| win | Left anterior insula | r = .047  *p* = 0.847 | r = .041  *p* = 0.866 | r = -.164  *p* = 0.501 | r = .412  *p* = 0.08(*) |
| lose | Right PACC | r = -.126  *p* = 0.609 | r = .028  *p* = 0.909 | r = -.273  *p* = 0.258 | r = -.311  *p* = 0.195 |
| lose | Left anterior insula | r = -.231  *p* = 0.341 | r = -.228  *p* = 0.348 | r = .162  *p* = 0.507 | r = -.343  *p* = 0.150 |

Pearson correlation coefficients [r], significant correlations are labelled

(***p* < 0.01, **p* < 0.05, (*)p<0.1), *two-sided*

**Abbreviations:** NS: novelty seeking, HA: harm avoidance, RD: reward dependence, P: persistence, PACC: pregenual anterior cingulate cortex
